# Patients and Provider Experiences with Telemedicine for Follow-up Care in Military Settings

**DOI:** 10.1177/26924366251372619

**Published:** 2025-08-27

**Authors:** Vahe Heboyan, Gianluca De Leo

**Affiliations:** Health Management Economics and Policy, School of Public Health, Augusta University, Augusta GA, USA.

**Keywords:** military health system, patient perception, telemedicine, willingness-to-pay

## Abstract

**Background:**

Telemedicine offers patients and physicians the opportunity to advance health care delivery to active duty and retired military personnel. Assessing the efficacy and feasibility of performing post-surgery follow-up assessment through telemedicine at a large military hospital, while examining patient and provider experience and satisfaction with the telemedicine follow-up visit and estimating patients’ willingness-to-use and willingness-to-pay for telemedicine consultations may help to shape the future use of telemedicine in the military settings.

**Methods:**

We administered surveys to 96 unique patients who agreed to perform a follow-up telemedicine visit, rather than a traditional face-to-face visit, after undergoing a minor surgery at a large army military hospital in the southeast region of the United States. We also administered a survey to six physicians.

**Results:**

Most of the patients strongly agreed (86.5%) or somewhat agreed (10.8%) that their medical problems were adequately addressed during the telemedicine visit. Most physicians were either satisfied (7.5%) or extremely satisfied (75.5%) with the overall experience. Less than half of the patients were willing to use a similar telemedicine visit for a fee in the future and only two patients were willing to pay $100 for such visit.

**Conclusion:**

After minor surgery, follow-up telemedicine visits may be an effective, efficient, and convenient alternative to face-to-face visits for active duty and retired military personnel.

## Introduction

Post-operation telemedicine consultations have been found to decrease patient readmission rates, saving time and the burden of travel.^1^ A previous literature review study reported that telemedicine benefits preoperative assessment and diagnosis, evaluation after surgery, and follow-up visits.^2^ The convenience of accessing telemedicine consultations from the home or office saves time, decreases the number of missed appointments, and reduces the number of working days missed.^2^ A recent research study on patient and clinician experience with telemedicine reported that telemedicine visits can provide effective follow-up. The same article also highlighted the trend that patients are embracing telemedicine to a surprising degree, particularly for follow-up care.^3^ Another recent study reported that postoperative follow-up performed with a telemedicine system is safe and acceptable to patients and could be considered in patients undergoing uncomplicated benign general surgery.^4^ Telemedicine treatment for depression and post-traumatic stress disorder has been found to be highly successful and more cost-effective than optimized usual care.^5,6^ A research study examining the use of telemedicine during the COVID-19 pandemic for follow-up reported that telemedicine can safely and effectively be performed in selected surgical patients.^7^ Another study indicated that telemedicine is a feasible alternative to reduce personal outpatient appointments for cancer follow‐up.^8^ A recent study investigated the patients’ willingness-to-use (WTU) and willingness-to-pay (WTP) for telehealth services that target emergency departments.^9^

Connecting health care providers with patients in real-time through the patient’s personal communication device represents a significant advancement in health care delivery to active duty and retired military personnel. A very recent research study reported that telemedicine has the potential to transform the Military Health System (MHS) through improvements in cost, quality, access, and readiness.^10^ The MHS demonstrates significant capability in the use of telemedicine in deployed and operational environments.^10^ The proposed study complements this foundation by expanding telemedicine to serve patients already familiar with their health care providers through the integration of a more efficient means to execute follow-up visits.

While there is considerable literature on the efficacy and the adoption of telemedicine, there has been little work the patient WTU and WTP for telehealth services that target the MHS. The research reported here focuses on (1) assessing the efficacy and feasibility of performing post-surgery follow-up assessment through telemedicine at a large military hospital, (2) examining patient and provider experience and satisfaction with the telemedicine follow-up visit, and (3) estimating patients’ WTU and WTP for telemedicine consultations.

## Methods

To meet the objectives of this study, we recruited patients who intended to undergo a variety of elective outpatient surgeries such as lipoma removal, umbilical hernia repair, or laparoscopic cholecystectomy at the Dwight D. Eisenhower Army Medical Center (DDEAMC), where approximately 500 patients undergo such procedures annually.

Patients who were considered by the surgeon to be clinically suitable for telemedicine follow-up and had access to a smartphone or tablet with an internet connection and a functioning camera were given the opportunity for post-surgery follow-up 2 weeks after surgery via telemedicine as an alternative to the conventional in-person visit. The patient’s decision to follow up via telemedicine was reviewed by the surgeon after surgery to verify clinical suitability.

At the time of electing the preferred mode of follow-up care, the nurse coordinator provided the patients with a brochure describing the follow-up process via telemedicine, its advantages and disadvantages, technological requirements, and contact information. We used a questionnaire to (1) collect the demographic information of the patients, (2) examine the patients’ reasons for choosing either telemedicine or in-person visitation, and (3) assess their knowledge and perception of telemedicine.

On the day of surgery, prior to surgery, patients were provided with training that included the practice of using their communication device (e.g., smartphone or tablet) in a mock session that simulated a telemedicine session. The nurse coordinator worked with the patient to establish communication to a telemedicine session between the provider’s telemedicine station and the patient’s communication device, checked the video and audio quality, and assessed the ability of the patient to conduct the mock telemedicine session. Upon completing this training, we used a questionnaire to assess patient perceptions and comfort toward utilizing telemedicine for post-surgery follow-up. Patients who reported feeling uncomfortable using telemedicine were referred for in-person follow-up.

At the 2-week follow-up session, the nurse coordinator established the connection with the patient and re-tested the quality of the connection. The surgeon instructed the patient on how to position their camera device such that it was possible to assess the progress of healing of the surgical wound. The surgeon also examined the site for any signs of potential infection or other aberrant surgical complications. After this session, based on the progress of healing of the surgical wound, the surgeon made the decision if the patient had to come back for an in-person follow-up visit. Upon completing the virtual follow-up, the patient and surgeon were asked to self-report their experience using a questionnaire. More specifically, we assessed the patient’s (1) level of confidence, comfort, and overall satisfaction with the telemedicine session experience and interaction with the surgeon, (2) unmet needs of the patient, as well as (3) the effectiveness of the telemedicine encounter, as determined by the surgeon and patient.

We also estimated the patient WTU and WTP for receiving post-surgery follow-up via telemedicine instead of receiving consultation in person. WTP is an economic valuation method that elicits respondents’ maximum WTP for certain goods and services. To measure the benefits of eHealth, WTP is a commonly used metric.^11,12^ We set the cost of services at a starting amount of $50 that, depending on patients’ willingness, was increased to $100 or decreased to $25. At the time of this project, we determined that several telemedicine services that allowed people to connect with a physician were available from $50 to $100 as an out-of-pocket expense.

We obtained IRB approval from the Eisenhower Army Medical Center.

## Results

A total of 104 patients signed the consent form. However, only 96 patients agreed to take part in our study. Among the eight patients who decided not to participate in our study, seven were female, three reported being white, five reported being black or African American, four were younger than 35 years old, one was between 36 and 50, and three were older than 51. Four patients traveled less than 10 miles, and four subjects traveled between 11 and 20 miles for their appointment. The main reasons for not being interested in conducting the post-surgical follow-up visit via teleconference were feeling uncomfortable communicating using the telemedicine technology (*n* = 3), preferring a face-to-face visit (3), and easiness to make the appointment in person (*n* = 1) because worked in the hospital. One patient reported feeling unbothered by attending the post-surgical follow-up visit in person.

Among the 96 patients who agreed to participate in this study, there were more males (*n* = 54) than females (*n* = 42). Approximately half of the patients reported a white, non-Hispanic race (*n* = 51). The three age brackets and the three income brackets were almost uniformly distributed. More than half of the patients had less than a bachelor’s degree (*n* = 57). Less than half of the patients reported having no children (*n* = 41). Only 16 patients reported not working currently. One-third of the patients reported being active-duty personnel, one-third were veterans or retirees, and one-third were civilian dependents. The two most common surgeries they had were laparoscopic inguinal hernia (*n* = 23) and umbilical hernia repair (*n* = 16). Approximately half of the patients (*n* = 52) waited up to 30 min from when they arrived at the hospital to when they completed the appointment. Only 17 patients waited more than 90 min. Approximately half of the patients (*n* = 49) drove less than 10 miles to the hospital. More than two-thirds of the patients (*n* = 73) reported living locally. Table 1 shows the patients’ demographics and the characteristics of their visits.

**Table 1.** Patient’s Demographics and Today Visit Characteristics

	Patients (*N* = 96)
Patient’s demographics	
Gender	
Male	54 (56.3%)
Female	42 (43.8%)
Age (years)	
18–35	35 (36.5%)
36–50	35 (36.5%)
51 and over	26 (27.1%)
Race	
White, non-Hispanic	51 (53.1%)
Black or African American	33 (34.4%)
Other	12 (12.5%)
Highest level of school obtained	
Less than bachelor’s degree	57 (59.4%)
Bachelor’s degree	21 (21.9%)
Master’s degree or higher	18 (18.8%)
Married status	
Married	74 (77.1%)
Not married	22 (22.9%)
Children less than 18 years of age live in same household	
0	41 (42.7%)
≥1	55 (57.3%)
Household income	
<60 K	35 (36.5%)
60–100 K	34 (35.4%)
>100 K	27 (28.1%)
Employment status	
Full time	37 (38.5%)
Military active	34 (35.4%)
Retired	9 (9.4%)
Not working	16 (16.7%)
Current military status	
Active duty	35 (36.5%)
Reserves	2 (2.1%)
National guard	2 (2.1%)
Veteran or retiree	29 (30.2%)
Civilian dependent	28 (29.2%)
Today Visit Characteristics	
Surgery/procedure	
Laparoscopic inguinal hernia	23 (24.0%)
Umbilical hernia repair	16 (16.7%)
Laparoscopic cholecystectomy (gallbladder removal)	15 (15.6%)
Lipoma removal	15 (15.6%)
Colonoscopy	6 (6.3%)
Cyst removal	5 (5.2%)
Open inguinal hernia	4 (4.2%)
Other	12 (12.5%)
Minutes passed from the time today arrival to the hospital and the time your appointment with the physician was completed	
Up to 30 min	52 (54.2%)
30–90	27 (28.1%)
>90	17 (17.7%)
Miles traveled today to reach the hospital	
<10	49 (51.0%)
11–20	27 (28.1%)
>20	20 (20.8%)
Location of stay in the area	
Friends/hotel	2 (2.1%)
Did not stay (single day or another reason to stay in the area)	21 (21.9%)
Live in the area	73 (76.0%)

More than half of the patients (*n* = 61) reported not hearing about telemedicine. Among the 35 patients who reported knowing about telemedicine, only five noted having used telemedicine in the past. Based on a 10-point scale where 0 meant not knowing telemedicine at all and 10 meant being very knowledgeable about telemedicine, more than half of the patients (*n* = 52) reported 4 or less, with one-third of the patients (*n* = 39) reporting 0. Only 6 patients reported being very knowledgeable.

Since three people withdrew from the procedure, only 93 patients participated in the mockup session with the nurse. A large majority of patients (*n* = 78) were either satisfied or extremely satisfied with the following aspects of the telemedicine session: audio, visual, personal comfort during the session, ease of use of technology, and overall experience. Only six patients were extremely dissatisfied with all the aspects. At the end of the mockup session, all 93 patients were still interested in using telemedicine for the follow-up visit. However, 21 patients did not attend the telemedicine follow-up visit: 14 patients canceled, six patients returned to the hospital for a face-to-face visit, and one patient was asked to return for an in-person follow-up. After the telemedicine follow-up visit, only 37 patients took the time to answer the questionnaires assessing the follow-up telemedicine visit with the physician. A vast majority of patients were either satisfied or extremely satisfied with the following aspects of the telemedicine session: audio, visual, personal comfort during the session, ease of connecting to the session, thoroughness, carefulness, and skillfulness of the telemedicine physician, how well the physician answered questions, the courtesy, respect, sensitivity, and friendliness of the telemedicine physician and overall experience. The aspect patients were mostly dissatisfied with was the easiness of connecting to the session. Table 2 shows the details of the patient’s satisfaction with the mockup session.

**Table 2.** Patient Experience with Telemedicine

Patient satisfaction with mockup and follow-up telemedicine visit					
	Extremely dissatisfied	Somewhat dissatisfied	Neither satisfied nor dissatisfied	Somewhat satisfied	Extremely satisfied
How satisfied were you with the following aspects of the mockup telemedicine session?					
Audio	6 (6.5%)	0 (0%)	9 (9.7%)	14 (15.1%)	64 (68.8%)
Visual	6 (6.5%)	0 (0%)	5 (5.4%)	18 (19.4%)	64 (68.8%)
Personal comfort during the session	6 (6.5%)	1 (1.1%)	6 (6.5%)	14 (15.1%)	66 (71.0%)
Ease of use of technology	6 (6.5%)	2 (2.2%)	7 (7.5%)	12 (12.9%)	66 (71.0%)
Overall experience	6 (6.5%)	0 (0%)	6 (6.5%)	10 (10.8%)	71 (76.3%)
How satisfied were you with the following aspects of the telemedicine session?					
Audio quality	0 (0%)	1 (2.7%)	0 (0%)	8 (21.6%)	28 (75.7%)
Visual quality	0 (0%)	1 (2.7%)	1 (2.7%)	9 (24.3%)	26 (70.3%)
Personal comfort during the session	0 (0%)	0 (0%)	0 (0%)	6 (16.2%)	31 (83.8%)
Ease of connecting to the session	1 (2.7%)	1 (2.7%)	1 (2.7%)	6 (16.2%)	28 (75.7%)
Thoroughness, carefulness, and skillfulness of the telemedicine physician	0 (0%)	0 (0%)	0 (0%)	3 (8.1%)	34 (91.9%)
Courtesy, respect, sensitivity, and friendliness of the telemedicine physician	0 (0%)	0 (0%)	0 (0%)	1 (2.7%)	36 (97.3%)
How well the physician answered your questions	0 (0%)	0 (0%)	0 (0%)	3 (8.1%)	34 (91.9%)
Overall experience	0 (0%)	0 (0%)	1 (2.7%)	3 (8.1%)	33 (89.2%)

Patient feedback on follow-up telemedicine visit					
	Strongly disagree	Somewhat disagree	Neither agree nor disagree	Somewhat Agree	Strongly agree
How much do you agree or disagree with the following?					
I was able to communicate adequately with the physician/health care provider.	0 (0%)	0 (0%)	0 (0%)	6 (16.2%)	31 (83.8%)
I was comfortable that the physician was able to understand my problem.	0 (0%)	0 (0%)	0 (0%)	4 (10.8%)	33 (89.2%)
Telemedicine made it easier for me to speak to the physician today.	0 (0%)	2 (5.4%)	1 (2.7%)	8 (21.6%)	26 (70.3%)
I would have received better care if I had seen the provider in person.	11 (29.7)	3 (8.1%)	11 (29.7)	4 (10.8%)	8 (21.6%)
Next time, I would prefer to see the physician in person despite the possible inconvenience.	11 (29.7)	6 (16.2%)	8 (21.6%)	3 (8.1%)	9 (24.3%)
Overall, I was satisfied with today’s telemedicine session.	0 (0%)	0 (0%)	2 (5.4%)	6 (16.2%)	29 (78.4%)
The telemedicine follow-up session was comfortable for me.	0 (0%)	0 (0%)	2 (5.4%)	3 (8.1%)	32 (86.5%)
I had difficulty hearing or understanding the physician over the Telemedicine system.	27 (73.0%)	3 (8.1%)	2 (5.4%)	2 (5.4%)	3 (8.1%)
I had difficulty seeing the physician over the Telemedicine system.	30 (81.1%)	3 (8.1%)	1 (2.7%)	2 (5.4%)	1 (2.7%)
I feel that my medical problem was adequately addressed in this Telemedicine visit.	0 (0%)	0 (0%)	1 (2.7%)	4 (10.8%)	32 (86.5%)

Most of the patients either strongly agreed or somewhat agreed that they were able to communicate adequately with the physician/health care provider, they were comfortable that the physician was able to understand their problems, they felt that their medical problems were adequately addressed in the telemedicine visit, that telemedicine made it easier to speak to the physician and that the telemedicine follow-up session was comfortable. Most of the patients either strongly disagreed or somewhat disagreed that they had difficulty hearing, understanding, or seeing the physician over the telemedicine system. Patients did not agree with the fact that they would have received better care if they had seen the provider in person and the fact that they would prefer to see the physician in person for a future visit despite the possible inconvenience. Overall, patients were satisfied with the follow-up telemedicine visit. A detailed report on the feedback received by the patients on the follow-up telemedicine visit is presented in Table 2.

Only one patient reported that the telemedicine follow-up visit did not save travel and waiting time compared to a face-to-face visit. Only two patients reported not wanting to use and four patients reported not being sure about using telemedicine for a similar post-surgical follow-up in the future. A large majority would recommend telemedicine for a similar post-surgical follow-up to others (*n* = 32). Only one patient would not recommend telemedicine. Almost half of the patients (*n* = 18) thought that compared to other health care visits in person, the time the physician spent via telemedicine was the same. Only 3 patients thought the time spent on telemedicine was greater, while the remaining 16 patients reported the time spent on telemedicine being less than the visit in person. More than half of the patients (*n* = 23) used a smartphone (e.g., iPhone, Samsung Galaxy, etc.) to connect to the telemedicine visit. Six patients used a tablet (e.g., iPad), and seven a laptop/desktop. One patient did not answer the question about the device used.

**FIG. 1.**
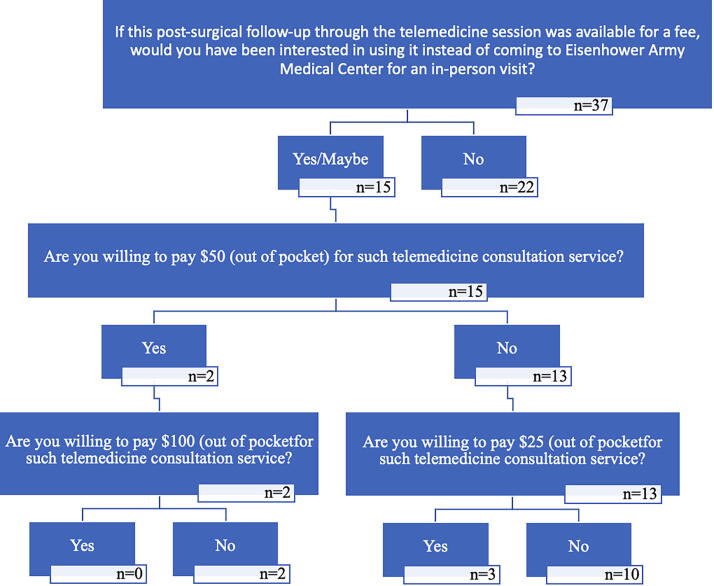
Willingness-to-Pay study diagram and respondent responses.

After introducing the patients to a hypothetical scenario where they could use a telemedicine follow-up visit for a fee instead of attending a face-to-face visit at the hospital, 15 (40.5%) of the patients reported they either would use or maybe use such telemedicine consultation, 22 (59.5%) would not. None of the patients were willing to pay $100 for the telemedicine consultation, 2 (0.05%) were willing to pay $50, 3 (0.8%) were willing to pay $25. Figure 1 shows the diagram of the question and the patients’ responses.

Six physicians completed 53 questionnaires about their satisfaction with follow-up telemedicine visits. They also provided their feedback. Two different physicians provided follow-up for one patient. Most physicians were either satisfied or extremely satisfied with the following aspects of the telemedicine session: audio quality, visual quality, ease of connecting to the session, and overall experience. Most physicians either strongly agreed or agreed that they were able to communicate adequately with the patients. Physicians reported having problems with communication three times: once the connection was dropped and required reconnecting, once the patient was unable to connect to the telemedicine service and the physician decided to perform the follow-up visit using a phone line without video capabilities, and once the physician reported seeing a slightly dark picture of the patient. Most physicians either strongly disagreed or disagreed that the telemedicine visit was uncomfortable for them and that they would prefer to see the patient in person despite the possible inconvenience for future visits. A detailed report on the satisfaction of the physicians on the follow-up telemedicine visit and their feedback is presented in Table 3.

**Table 3.** Physician Satisfaction with and Feedback on Follow-up Telemedicine Visit

	Extremely dissatisfied	Somewhat dissatisfied	Neither satisfied nor dissatisfied	Somewhat satisfied	Extremely satisfied
How satisfied were you with the following aspects of the telemedicine session?					
Audio quality	3 (5.7%)	2 (3.8%)	4 (7.5%)	2 (3.8%)	42 (79.2%)
Visual quality	3 (5.7%)	2 (3.8%)	5 (9.4%)	6 (11.3%)	37 (69.8%)
Ease of connecting to the session	3 (5.7%)	1 (1.9%)	3 (5.7%)	3 (5.7%)	43 (81.1%)
Overall experience	3 (5.7%)	0 (0%)	6 (11.3%)	4 (7.5%)	40 (75.5%)

	Strongly disagree	Somewhat disagree	Neither agree nor disagree	Somewhat Agree	Strongly agree
I was able to communicate adequately with the patient	1 (1.9%)	2 (3.8%)	3 (5.7%)	3 (5.7%)	44 (83.0%)
The telemedicine visit was uncomfortable for me	49 (92.5%)	3 (5.7%)	1 (1.9%)	0 (0%)	0 (0%)
Next time, I would prefer to see the patient in person despite the possible inconvenience	45 (84.9%)	7 (13.2%)	1 (1.9%)	0 (0%)	0 (0%)

All 53 surveys from the six physicians reported that in the future, they would use telemedicine for a similar post-surgical follow-up with other patients. There were only two occasions in which the physicians reported being unable to make an accurate assessment through the telemedicine session. In one case, the telemedicine system did not connect at all, and in the other case, the physician noted redness around the incision that could have been a sign of deeper infection. The report on the outcome of the follow-up was: The patient is healing and progressing well (*n* = 51), the patient experienced issues or complications that will require further follow-up (*n* = 2), and I could not make an accurate assessment (*n* = 0). Following the telemedicine visits, only three patients were recommended an in-person follow-up appointment by their physicians. All post-operative issues were adequately addressed in the telemedicine visit.

## Discussion

This research projects investigates aims to assess the efficacy and feasibility of performing post-surgery follow-up assessment through telemedicine at a large military hospital. The views of both patients and clinicians have been considered. Based on our knowledge, our project is the first to try to estimate the WTU and WTP for telemedicine services among patients who use the MHS.

We designed our study first with a mock session that simulated a telemedicine session to decrease the possibility patients would cancel their telemedicine visit due to technical difficulties. Also, at the 2-week follow-up session, the nurse coordinator established the connection with the patient and re-tested the quality of the connection prior of the encounter with the physician. We believe this approach helped our patients to fully understand the telemedicine visit option and helped them to have a positive follow-up visit.

Patient satisfaction for follow-up visits is compromised largely by the commitment of time and money required to travel to the clinic, process through the receiving area, and wait on the availability of the providers. Associated with the travel time are the cost of transportation, forgone income, inconvenience of travel and waiting, and the effect on the environment and traffic patterns of what may now be evolving as the unnecessary use of roadways. Often, scheduling a follow-up appointment requires taking time off work for a visit that amounts to a relatively brief encounter with the provider to assess wound progress and general surgical recovery.^13^ Most of our patients were either somewhat satisfied or extremely satisfied with the telemedicine visit. However, slightly less than one third of patients said they would like to see the physician in person at the next appointment despite the possible inconvenience. Also, the physicians involved in our study showed they were generally satisfied with the telemedicine system. In a previous research study focused on hip and knee replacements, the use of a virtual clinic was well accepted by both patients and clinicians.^14^ Another study reported that providers were satisfied with a virtual visit for long-term follow-up care.^15^

In our study, less than half of the patients were willing to use a similar telemedicine visit for a fee in the future and only two patients were willing to pay $100 for such visit. A recent systematic review on Willingness to Pay for telemedicine services among patients with chronic diseases ranged from 19% to 70% across the studies.^16^ The WTP questions are asked to solicit how much of economic/monetary value a respondent places for services or products that do not exist or are not offered yet. In our study, the telemedicine service for post-surgical follow-up visits was not offered by DDEAMC. We wanted to understand what economic or monetary value does the patient attach to the service received. The results can used by decision makers and policymakers to better understand the pricing and fee structures. Although currently the military pays for service member (SM) health care, hypothetical studies like ours are designed to provide information in case of policies change. For example, should military no longer be willing to pay for services or decide to make changes to its health care offering, studies like our will be used to better understand how SMs will perceive the new policy.

Our study has limitations. We only reported on patients who used the telemedicine system rather than investigating the perception of using telemedicine among all the patients of the hospital. Not all the physicians at the hospital took part in this project. This sample is not large enough to allow for generalization. Most patients lived close to the hospital facility, therefore their willingness to use and pay may have been biased.

Future studies should compare the patients and providers satisfaction of the in-person consults with the telemedicine consults and use a focus group to investigate in detail the patients’ motivations to not engage with the telemedicine session.

Telemedicine is a compelling frontier within surgery because of its potential to replace in-person clinical visits with virtual visits for post-surgical patient follow-up. Telemedicine has the potential to save both time and money while providing a satisfactory service to patients and providers, allowing surgeons to perform an essential component of the post-surgery exam efficiently. Improved access to care translates into improved readiness of SMs. The reduced time required for SMs to conduct in-person follow-up appointments would allow them to commit to continued military training and preparation.

## Abbreviations Used

AMEDD: U.S. Army Medical Department
AMTI: Advanced Medical Technology Initiative
DDEAMC: Dwight D. Eisenhower Army Medical Center
MHS: Military Health System
SM: Service member
WTP: Willingness-to-pay
WTU: Willingness-to-use

## References

[1] Augestad KM, Sneve AM, Lindsetmo RO. Telemedicine in postoperative follow-up of STOMa PAtients: A randomized clinical trial (the STOMPA trial). Br J Surg 2020;107(5):509–518; doi: 10.1002/bjs.11491 32100297

[2] Asiri A, AlBishi S, AlMadani W, et al. The use of telemedicine in surgical care: A systematic review. Acta Inform Med 2018;26(3):201–206; doi: 10.5455/aim.2018.26.201-206 30515013 PMC6195401

[3] Donelan K, Barreto EA, Sossong S, et al. Patient and clinician experiences with telehealth for patient follow-up care. Am J Manag Care 2019;25(1):40–44. 30667610

[4] Fink T, Chen Q, Chong L, et al. Telemedicine versus face-to-face follow up in general surgery: A randomized controlled trial. ANZ J Surg 2022;92(10):2544–2550; doi: 10.1111/ans.18028 36069322 PMC9826044

[5] Engel CC, Jaycox LH, Freed MC, et al. Centrally assisted collaborative telecare for posttraumatic stress disorder and depression among military personnel attending primary care: A randomized clinical trial. JAMA Intern Med 2016;176(7):948–956; doi: 10.1001/jamainternmed.2016.2402 27294447

[6] Lavelle TA, Kommareddi M, Jaycox LH, et al. Cost-effectiveness of collaborative care for depression and PTSD in military personnel. Am J Manag Care 2018;24(2):91–98. 29461856

[7] Irarrazaval MJ, Inzunza M, Munoz R, et al. Telemedicine for postoperative follow-up, virtual surgical clinics during COVID-19 pandemic. Surg Endosc 2021;35(11):6300–6306; doi: 10.1007/s00464-020-08130-1 33140151 PMC7605475

[8] Sonagli M, Cagnacci Neto R, Leite FPM, et al. The use of telemedicine to maintain breast cancer follow-up and surveillance during the COVID-19 pandemic. J Surg Oncol 2021;123(2):371–374; doi: 10.1002/jso.26327 33333581

[9] Heboyan V, Coule P, Mariotti D, et al. Knowledge, readiness, willingness-to-use, and willingness-to-pay for telehealth in nonlife-threatening emergency department visits. Telemed Rep 2025;6(1):34–43; doi: 10.1089/tmr.2024.0085 39991644 PMC11839518

[10] Madsen C, Poropatich R, Koehlmoos TP. Telehealth in the military health system: Impact, obstacles, and opportunities. Mil Med 2023;188(Suppl 1):15–23; doi: 10.1093/milmed/usac207 36882030

[11] Olsen JA, Smith RD. Theory versus practice: A review of ‘willingness-to-pay’ in health and health care. Health Econ 2001;10(1):39–52; doi: 10.1002/1099-1050(200101)10:1<39::aid-hec563>3.0.co;2-e 11180568

[12] Robinson R. Cost-benefit analysis. Bmj 1993;307(6909):924–926; doi: 10.1136/bmj.307.6909.924 8241859 PMC1679054

[13] Kane LT, Thakar O, Jamgochian G, et al. The role of telehealth as a platform for postoperative visits following rotator cuff repair: A prospective, randomized controlled trial. J Shoulder Elbow Surg 2020;29(4):775–783; doi: 10.1016/j.jse.2019.12.004 32197766

[14] Parkes RJ, Palmer J, Wingham J, et al. Is virtual clinic follow-up of hip and knee joint replacement acceptable to patients and clinicians? A sequential mixed methods evaluation. BMJ Open Qual 2019;8(1):e000502; doi: 10.1136/bmjoq-2018-000502 PMC6567956 31259271

[15] Kenney LB, Vrooman LM, Lind ED, et al. Virtual visits as long-term follow-up care for childhood cancer survivors: Patient and provider satisfaction during the COVID-19 pandemic. Pediatr Blood Cancer 2021;68(6):e28927; doi: 10.1002/pbc.28927 33559385 PMC7995169

[16] Chua V, Koh JH, Koh CHG, et al. The willingness to pay for telemedicine among patients with chronic diseases: Systematic review. J Med Internet Res 2022;24(4):e33372; doi: 10.2196/33372 35416779 PMC9047785

